# First-Trimester Crown-Rump Length and Embryonic Volume of Fetuses with Structural Congenital Abnormalities Measured in Virtual Reality: An Observational Study

**DOI:** 10.1155/2017/1953076

**Published:** 2017-03-21

**Authors:** L. Baken, B. Benoit, A. H. J. Koning, P. J. van der Spek, E. A. P. Steegers, N. Exalto

**Affiliations:** ^1^Department of Obstetrics and Gynecology, Division of Obstetrics and Prenatal Medicine, Erasmus MC, University Medical Center Rotterdam, Rotterdam, Netherlands; ^2^Department of Obstetrics and Gynecology, Princess Grace Hospital, Monaco-Ville, Monaco; ^3^Department of Bioinformatics, Erasmus MC, University Medical Center Rotterdam, Rotterdam, Netherlands

## Abstract

*Background.* With the introduction of three-dimensional (3D) ultrasound it has become possible to measure volumes. The relative increase in embryonic volume (EV) is much larger than that of the crown-rump length (CRL) over the same time period. We examined whether EV is a better parameter to determine growth restriction in fetuses with structural congenital abnormalities.* Study Design, Subjects, and Outcome Measures.* CRL and EV were measured using a Virtual Reality (VR) system in prospectively collected 3D ultrasound volumes of 56 fetuses diagnosed with structural congenital abnormalities in the first trimester of pregnancy (gestational age 7^+5^ to 14^+5^ weeks). Measured CRL and EV were converted to *z*-scores and to percentages of the expected mean using previously published reference curves of euploid fetuses. The one-sample *t*-test was performed to test significance.* Results.* The EV was smaller than expected for GA in fetuses with structural congenital abnormalities (−35%  *p* < 0.001, *z*-score −1.44  *p* < 0.001), whereas CRL was not (−6.43%  *p* = 0.118, *z*-score −0.43  *p* = 0.605).* Conclusions.* CRL is a less reliable parameter to determine growth restriction in fetuses with structural congenital abnormalities as compared with EV. By measuring EV, growth restriction in first-trimester fetuses with structural congenital abnormalities becomes more evident and enables an earlier detection of these cases.

## 1. Introduction

In the past decade prenatal screening has partly shifted from the second trimester to the first trimester of pregnancy. Because of vast improvements in imaging technology the embryo and fetus in early pregnancy can be evaluated in much more detail, allowing screening for structural abnormalities between 11 and 14 weeks GA [1–5]. A significant proportion of major structural abnormalities can be detected already in this period. In some cases, nonspecific findings, like increased nuchal translucency, may be the first sign for existing structural abnormalities, leading to additional ultrasound examinations [6].

It is well known that first-trimester growth is associated with pregnancy outcome [7–10] and that several factors like maternal factors and dietary pattern influence first-trimester growth [11–13]. Traditionally, first-trimester fetal growth has been documented by two-dimensional (2D) crown-rump length (CRL) measurements. With the introduction of three-dimensional (3D) ultrasound it has become possible to measure embryonic volumes (EV) [14]. Earlier studies show that the relative increment of the EV is much larger than the increment of the CRL during the same period [15]. Using an innovative 3D Virtual Reality (VR) technique, Rousian et al. demonstrated in this study that when the CRL doubles the EV increases 6.5-fold. Volume measurement might therefore enable earlier detection of fetal growth restriction in pregnancy. It is well known that too small CRL is a clinical predictor for miscarriage, chromosomal abnormalities (especially trisomy 18), and fetal growth restriction in the second and third trimester of pregnancy [10, 16–19]. It has been suggested that EV is smaller in aneuploid pregnancies and by using VR it was proven that, compared with CRL, EV was not only smaller in trisomy 18 pregnancies but also in trisomy 21 and trisomy 13 pregnancies [20, 21]. EV therefore turns out to be a better parameter to detect growth restriction caused by aneuploidy than CRL.

From these observations it is suspected that underlying pathophysiological changes in these cases might influence embryonic and early fetal growth. First-trimester growth might also be impaired in pregnancies diagnosed with a congenital abnormality. An association between the presence of structural congenital abnormalities and second- and third-trimester growth restriction is already known for a long time [22–24].

The aim of this study is to examine the first-trimester growth pattern in embryos and fetuses with structural congenital abnormalities. CRL and EV measurements of pregnancies with structural abnormalities were compared with references values of CRL and EV in uncomplicated pregnancies.

## 2. Methods

Between December 2008 and November 2013 transvaginal three-dimensional (3D) ultrasound volumes were collected of first-trimester pregnancies in which a structural congenital abnormality was diagnosed (*N* = 71). Cases were collected at the department of Obstetrics and Prenatal Medicine at Erasmus MC University Medical Center Rotterdam (*n* = 47) and at Hôpital Princesse Grace Monaco (*n* = 15). Additional cases (*N* = 9) were included from the Rotterdam Predict study [11], a periconception cohort aimed at early pregnancy. Ultrasound scans were performed using the Voluson E8 Expert system (GE Medical Systems, Zipf, Austria) by operators experienced in collecting 3D ultrasound datasets. Structural congenital abnormalities were all confirmed either during the midpregnancy ultrasound scan, postpartum diagnosis, or a pathological investigation after termination of pregnancy.

In spontaneously conceived pregnancies dating was based on the first day of the last menstrual period (LMP). When the menstrual cycle was regular but >3 days different from 28 days the gestational age (GA) was adjusted for the cycle length. In pregnancies conceived by in vitro fertilization (IVF) with or without intracytoplasmic sperm injection (ICSI) GA was calculated from the day of oocyte retrieval plus 14 days. In pregnancies originating from intrauterine insemination GA was calculated based on the LMP or inseminated date plus 14 days. If the first day of the LMP was missing or if the menstrual cycle was irregular, these pregnancies were excluded from this analysis. The GA ranged from 7^+5^ to 14^+5^ weeks.

**Figure 1:**
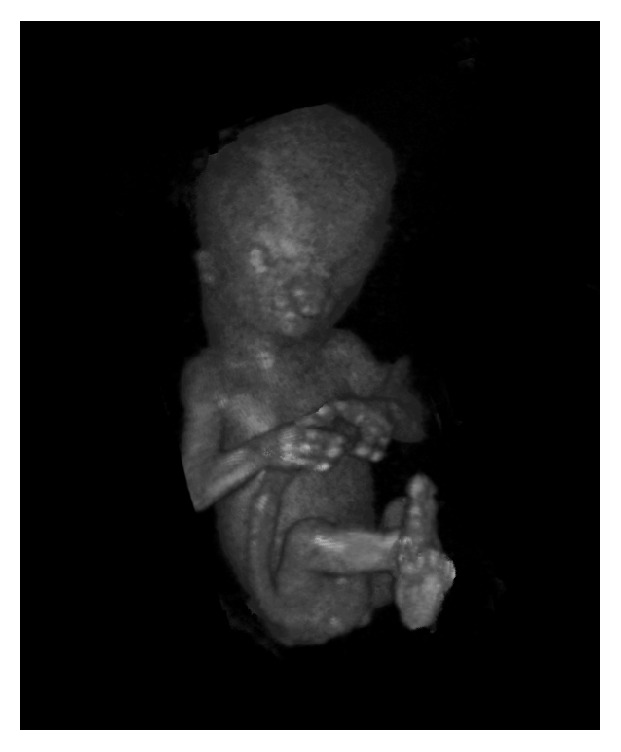
3D transvaginal ultrasound dataset of a fetus with an ectrodactyly ectodermal dysplasia-cleft (EEC) syndrome visualized in Virtual Reality. Bilateral split hands and split feet are seen as well as bilateral cheilognatoschisis. An overriding aorta with a ventricle septum defect was diagnosed additionally.

The 3D volumes were converted to Cartesian volumes, using 3D software (4D View, GE Medical Systems, Zipf, Austria), and transferred to the BARCO (Kortrijk, Belgium) I-Space VR system at the department of Bioinformatics of Erasmus MC University Medical Center Rotterdam. This is a four-walled CAVE™ like VR system in which investigators are surrounded by stereoscopic images. A “hologram” of the ultrasound data is created by the V-Scope [25] volume rendering application (Erasmus MC, Rotterdam, the Netherlands) and polarized glasses enable the viewer to perceive depth and to interact with 3D volumes in an intuitive manner. In the I-Space all 3D ultrasound volumes were evaluated and the best volume for each case was selected based on image quality and completeness of the volume. A fetus with structural congenital abnormalities visualized in Virtual Reality is shown in Figure 1.

CRL and EV were measured in the BARCO I-Space using the V-Scope software. The V-Scope application includes a region-growing segmentation algorithm combined with a neighbourhood variation threshold for semiautomatica volume calculation in selected structures [25]. The procedure for measuring EV is described in detail by Rousian et al. [15] The innovative VR technique has already been successfully applied in various prenatal studies [14, 25].

To include all body parts of the embryo, the omphalocele, physiological or pathological, is included in the EV calculation, as well as hydrops, frequently present in fetuses with structural congenital abnormalities. All measurements were performed by the same investigator (LB). The accuracy and reproducibility of CRL and EV measurements have been proven in previous studies and CRL and EV reference curves have been established [15, 26–28].* Inter- and intraobserver variability for 3D-VR measurements were very high for CRL (ICC 1.000; 95%  CI: 0.999–1.000, resp., ICC 1.000; 95% CI: 0.999–1.000) as well as for EV measurements (ICC 0.999; 95% CI: 0.997–0.999, resp., 0.999; 95% CI: 0.998–0.999) [27, 29].* The data of the present study are compared with these reference curves.

This study has been approved by the Central Committee on Research in The Hague and the Local Medical Ethical and Institutional Review Board of the Erasmus MC (METC2004-227).

### 2.1. Statistical Analysis

In each pregnancy complicated by a congenital abnormality the observed values for CRL and/or EV were subtracted from the expected mean of CRL and EV for GA. This expected value was obtained from reference curves published in earlier studies [15, 26–29]. This difference was divided by the standard deviation (SD) for GA of the reference values in order to obtain the *z*-score. This difference was as well expressed as a percentage of the mean CRL and EV of reference fetuses. When different ultrasound volumes of different GA were present, the dataset of the oldest GA was used. The same analysis was performed when the expected value for EV was corrected for the measured CRL.

The one-sample two-sided  *t*-test was used to test for a statistically significant difference in *z*-score as compared to the reference value. This analysis was performed in the overall group of cases with structural congenital abnormalities and in the different subgroups of various structural congenital abnormalities.

Data analysis was performed using SPSS v.21 (SPSS Inc., Chicago, IL, USA). A *p* value < 0.05 was considered statistically significant.

## 3. Results

Three cases were excluded from the analysis because of uncertain GA and one because of a twin pregnancy. We excluded 11 cases for the measurements of both CRL and EV due to poor image quality caused by an intermediate position of the uterus or movement artifacts (*N* = 9), due to incompleteness of the volume (*N* = 1) and because of absence of heartbeat at the time of the ultrasound scan (*N* = 1). A total of 56 cases remained for analysis of CRL. As in five of these cases the image quality was too poor for performing EV measurement; only 51 cases remained for analysis of EV. Of these 7 of 56 cases were conceived by artificial reproductive techniques, 4 using ICSI, 2 using IVF, and 1 using IUI.

In the overall group of fetuses with structural congenital abnormalities the EV was smaller than expected for GA (−35%  *p* < 0.001, *z*-score −1.44 *p* < 0.001), whereas CRL was not smaller than expected (−6.43%  *p* = 0.118, *z*-score −0.43 *p* = 0.605). In 15 out of the 56 cases (26,8%) with structural abnormalities the CRL was more than two standard deviations below the mean (a *z*-score > −1,64). In 18 out of 51 cases (35,3%) the EV was more than two standard deviations below the mean. The CRL was significantly smaller in the subgroups with urogenital abnormalities and in the subgroup with hydropic abnormalities. The EV was significantly smaller in the subgroup with cardiac abnormalities, gastrointestinal abnormalities, urogenital abnormalities, neurological abnormalities, and in the group with hydropic abnormalities (Table 1).

**Figure 2:**
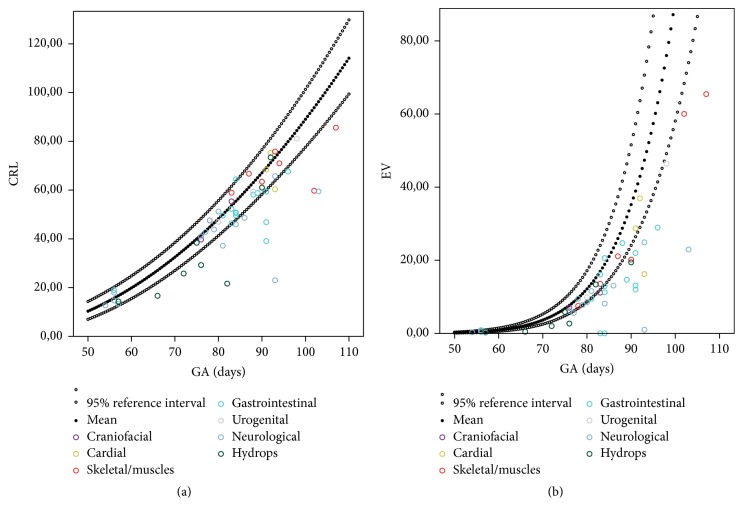
CRL (*n* = 56) and EV (*n* = 51) of all cases with structural congenital abnormalities plotted relative to the reference curves for healthy pregnancies.

In Figure 2 CRL and EV of all cases with structural congenital abnormalities are plotted in the reference curves for pregnancies without structural congenital abnormalities. In the supplemental figures the different groups of structural congenital abnormalities are plotted separately on the references curves for CRL and EV (Supplemental Figure 1 is available online at https://doi.org/10.1155/2017/1953076).

In Table 2 the percentage difference and *z*-score for observed versus expected EV after correction for the observed CRL is presented. No statistical differences were found.

## 4. Discussion

To the best of our knowledge this is the first study that investigates the relationship between EV and first-trimester structural congenital abnormalities. Although overall the CRL was not significantly smaller in fetuses with structural congenital abnormalities, a smaller than expected CRL was observed in hydropic fetuses and fetuses with urogenital abnormalities. In contrast to CRL, EV was statistically significant smaller than expected in the overall group of structural congenital abnormalities. In all subgroups, except for those with craniofacial and skeletal/muscle abnormalities, we found a significantly smaller EV than expected.

The mean difference in EV was more evident than the mean difference in CRL and went up to −43% (*z*-score −1.70) in fetuses with neurological abnormalities. This can be explained by the fact that a volume is a three-dimensional measurement in contrast to CRL, which is a flat, two-dimensional distance measurement. It was already demonstrated by Rousian et al. that when the CRL doubles EV increases 6.5 times [15]. However, after correcting the EV for the measured CRL significant differences were no longer present, suggesting proportional growth restriction. EV turned out to be a better parameter to detect first-trimester growth restriction as compared with CRL.

From the literature it has recently become evident that a detailed anatomical scan can be successfully preformed at the end of the first-trimester. The majority of major structural congenital abnormalities can therefore be diagnosed between 11 and 14 weeks GA. EV measurements can be performed from 6 weeks GA onwards [15] and may therefore possibly be used as a marker of an underlying abnormality long before an early anomaly scan can be performed. EV measurements in early pregnancy might point a clinician to the increased risk of a congenital abnormality. The effectiveness of EV as a marker for structural congenital abnormalities should be subject of further study.

The combination of early growth restriction and the presence of structural congenital abnormalities might be due to underlying pathological mechanisms. Growth restriction might either occur as a result of a structural congenital abnormality or growth restriction and structural congenital abnormalities might have a common etiological factor.

Limitations of the study are the low numbers of included cases with structural congenital abnormalities. Still finding significant differences for EV suggests a strong relationship of first-trimester structural congenital abnormalities and a decreased EV. Therefore, increasing the numbers in future studies will most likely only strengthen this relationship. Pregnancies with known chromosomal abnormalities were not included in the study. As fetal karyotyping was performed in 36 of 56 cases; it may be possible that cases with a chromosomal abnormality in our study group remained unnoticed. However, in all but 5 cases with an increased nuchal translucency, hygroma colli or hydrops fetalis, karyotyping was performed and showed to be euploid. The five cases with an increased nuchal translucency, hygroma colli or hydrops fetalis, were all in “hydrops” group and either spontaneously miscarried or were terminated before karyotyping could be performed.

We included pregnancies conceived by artificial reproductive techniques in our series of cases with structural congenital abnormalities. Recent studies point out that growth trajectories in early pregnancy do not differ between spontaneously conceived pregnancies and pregnancies conceived using artificial reproductive techniques in our population [30].

Furthermore, the BARCO I-Space is too large and too expensive to become a routine method for the measurement of EV. However, a much smaller and more affordable 3D VR desktop system is currently being evaluated and will provide a good alternative, making this technique broadly available to hospitals [31]. Following the introduction of the desktop VR system we foresee implementation of VR as an option in ultrasound machines in the near future.

We are aware that 3D ultrasound and its calculating software, that is, 4D view, are widely available for volume calculations, in contrast to the VR technique. However, using the available software on the ultrasound machine requires delineating the contours of the embryo manually in several different planes, which is subject to individual variation. The semiautomatic approach of the I-Space and its true depth perception allow for more objective volume measurements and prevent incomplete segmentations. Another advantage of the VR technique is that the whole body volume is measured with this technique, whereas when using the manual delineating technique only a head and trunk volume can be calculated, resulting in an underestimation.

## 5. Conclusions

In conclusion, CRL, the current golden standard for the detection of first-trimester growth restriction, seems a less reliable parameter to detect growth restriction in fetuses with structural congenital abnormalities as compared with EV, being significantly decreased in these pregnancies. By measuring EV, first-trimester growth restriction becomes more evident and might enable an earlier detection of cases at risk for a congenital abnormality.

## Supplementary Material

The individual CRL and EV measurements of the different groups of structural congenital abnormalities are plotted on the reference curves separately. This illustrates the the differences are more outspoken in the groups of gastrointestinal and neurological abnormalities as compared to the other groups.

## Figures and Tables

**Table 1:** Mean percentage difference and *z*-scores for CRL and EV in both the overall group of structural congenital abnormalities and in the various subgroups.

Variable/congenital abnormality	Mean difference in				
	*n*	% (95% CI)	*p* ^*∗*^	*z*-score (95% CI)	*p* ^*∗*^
*CRL*					
Overall	56	−6.43 (−14.55, 1.69)	0.118	−0.46 (−2.21, 1.30)	0.605
Craniofacial	2	−0.60 (−50.36, 49.15)	0.903	−0.05 (−6.25, 6.15)	0.931
Cardiac	5	−5.03 (−16.46, 6.39)	0.288	−1.34 (−2.27, 0.90)	0.296
Skeletal/muscles	8	−3.02 (−18.58, 12.54)	0.660	−0.55 (−2.62, 1.51)	0.547
Gastrointestinal	15	−7.02 (−18.05, 4.02)	0.194	−1.08 (−2.44, 0.28)	0.111
Urogenital	5	−4.98 (−7.87, −2.09)	0.009	−0.64 (−1.04, −0.25)	0.010
Neurological	14	0.12 (−30.53, 30.77)	0.993	1.59 (−5.74, 8.94)	0.646
Hydrops	7	−26.89 (−43.26, −8.51)	0.011	−2.94 (−5.18, −0.71)	0.018
*EV*					
Overall	51	−34.91 (−41.97, −27.85)	<0.001	−1.44 (−1.71, −1.16)	<0.001
Craniofacial	2	−21.22 (−187.3, 144.85)	0.351	−1.00 (−8.73, 6.73)	0.349
Cardiac	5	−31.86 (−56.43, −7.28)	0.023	−1.34 (−2.30, −0.39)	0.017
Skeletal/muscles	6	−26.18 (−60.67, 8.30)	0.108	−0.92 (−2.21, 0.37)	0.127
Gastrointestinal	13	−35.78 (−49.45, −22.11)	<0.001	−1.53 (−2.09, −0.96)	<0.001
Urogenital	4	−16.26 (−25.21, −7.31)	0.010	−0.75 (−1.22, −0.27)	0.016
Neurological	14	−43.24 (−59.20, −27.28)	<0.001	−1.70 (−2.27, −1.13)	<0.001
Hydrops	7	−40.85 (−66.34, −15.36)	0.008	−1.76 (−2.88, −0.64)	0.008

^*∗*^It is for observed mean difference versus 0.

**Table 2:** The mean percentage differences and *z*-scores for EV after correction for the observed CRL both in the overall group of structural congenital abnormalities and the subgroups of structural congenital abnormalities.

Variable/congenital abnormality	Mean difference in				
	*n*	% (95% CI)	*p* ^*∗*^	*z*-score (95% CI)	*p* ^*∗*^
*EV*					
Overall	51	27.86 (−14.34, 70.05)	0.191	0.92 (−0.66, 2.49)	0.247
Craniofacial	2	−6.18 (−266.27, 253.92)	0.813	−0.40 (−11.64, 10.84)	0.730
Cardiac	5	2.90 (−11.97, 17.76)	0.617	−0.10 (−0.71, 0.51)	0.673
Skeletal/muscles	6	29.69 (−71.29, 130.66)	0484	0.88 (−3.07, 4.82)	0.591
Gastrointestinal	13	14.94 (−4.72, 34.60)	0.124	0.47 (−0.38, 1.31)	0.254
Urogenital	4	4.95 (−2.93, 12.82)	0.139	0.008 (−27.34, 28.98)	0.932
Neurological	14	−1.82 (−25.66, 22.03)	0.872	−0.12 (−1.11, 0.87)	0.798
Hydrops	7	150.26 (−211.03, 511.54)	0.348	5.50 (−7.89, 18.89)	0.354

^*∗*^It is for observed mean difference versus 0.
