# Supplementary material for: First-Trimester Crown-Rump Length and Embryonic Volume of Fetuses with Structural Congenital Abnormalities Measured in Virtual Reality: An Observational Study
Source: Biomed Res Int. 2017 Mar 21;2017:1953076. doi: 10.1155/2017/1953076 (PMC5379074; doi:10.1155/2017/1953076)
Supplement: Supplementary file 1 — The individual CRL and EV measurements of the different groups of structural congenital abnormalities are plotted on the reference curves separately. This illustrates the the differences are more outspoken in the groups of gastrointestinal and neurological abnormalities as compared to the other groups. [file 1953076.f1.pdf]

Supplemental figure 1

The different groups of structural congenital abnormalities are plotted separately on the references curves for CRL and EV; craniofacial abnormalities (A), cardiac abnormalities (B), skeletal and/or muscle abnormalities (C), gastro-intestinal abnormalities (D), urogenital abnormalities (E), neurological abnormalities (F)and hydropic abnormalities (G).

A

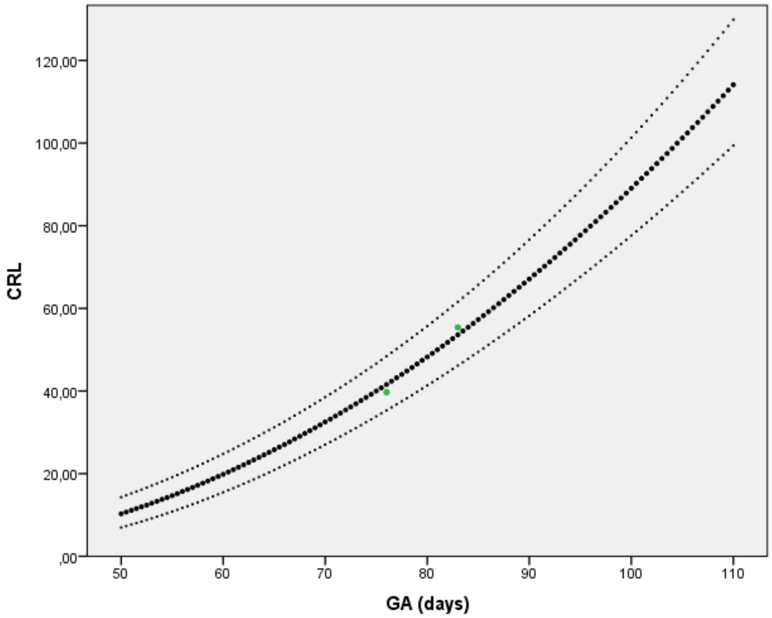

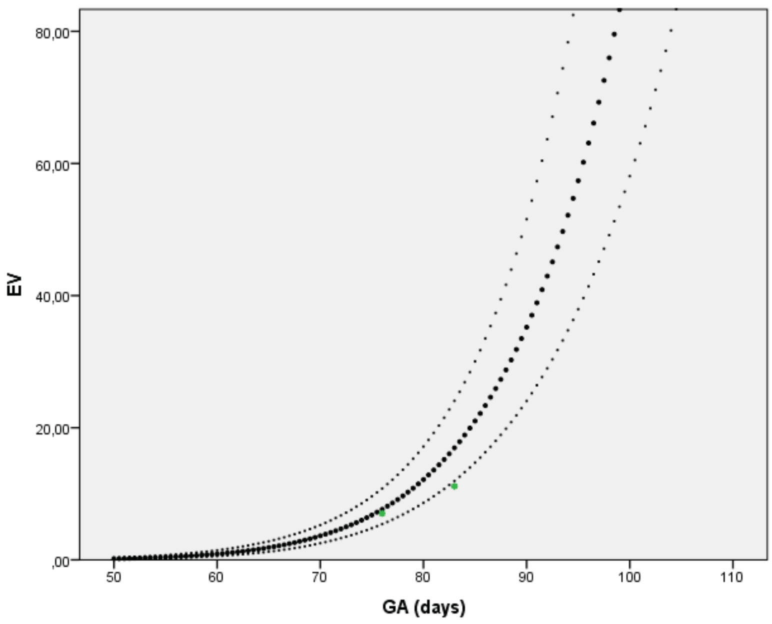
EV
80,00
60,00
40,00
20,00
,00
50
60
70
80
90
100
110
GA (days)

B

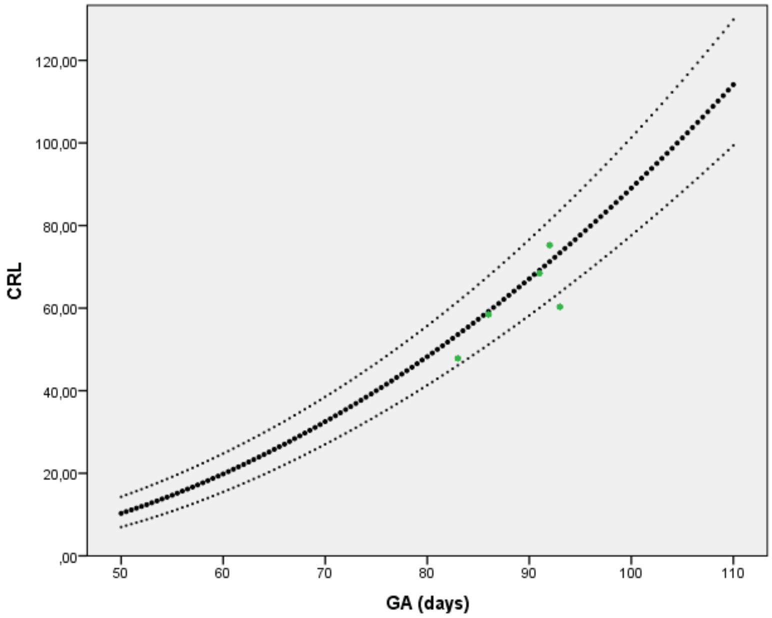
CRL
GA (days)
120,00
100,00
80,00
60,00
40,00
20,00
,00
50
60
70
80
90
100
110

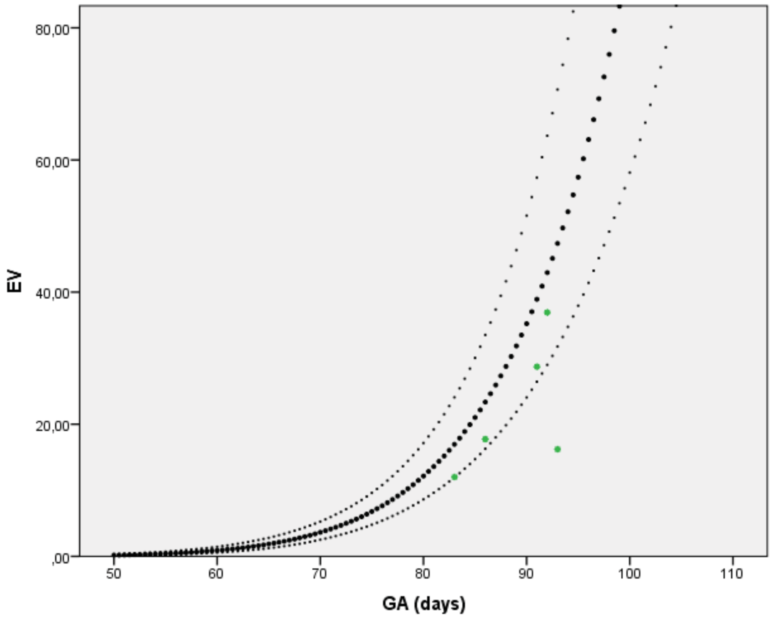
EV
GA (days)
80,00
60,00
40,00
20,00
,00
50
60
70
80
90
100
110

C

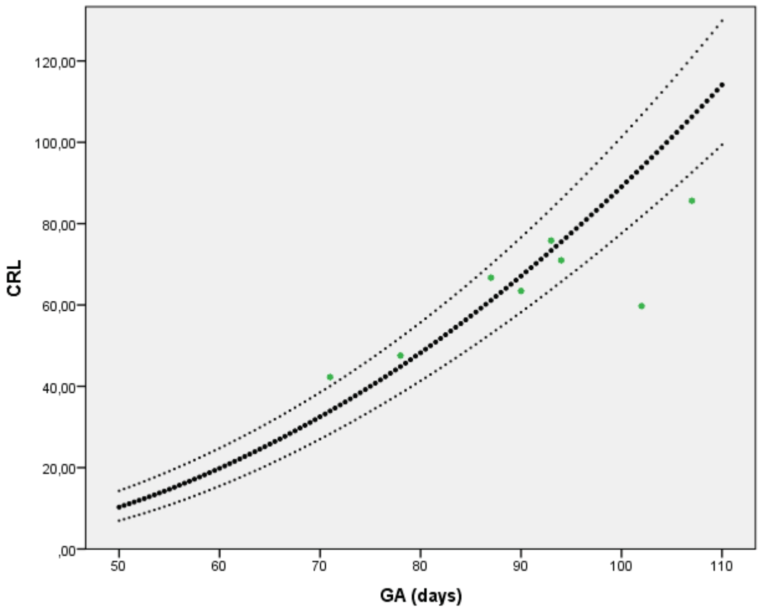
CRL
GA (days)
120,00
100,00
80,00
60,00
40,00
20,00
,00
50
60
70
80
90
100
110

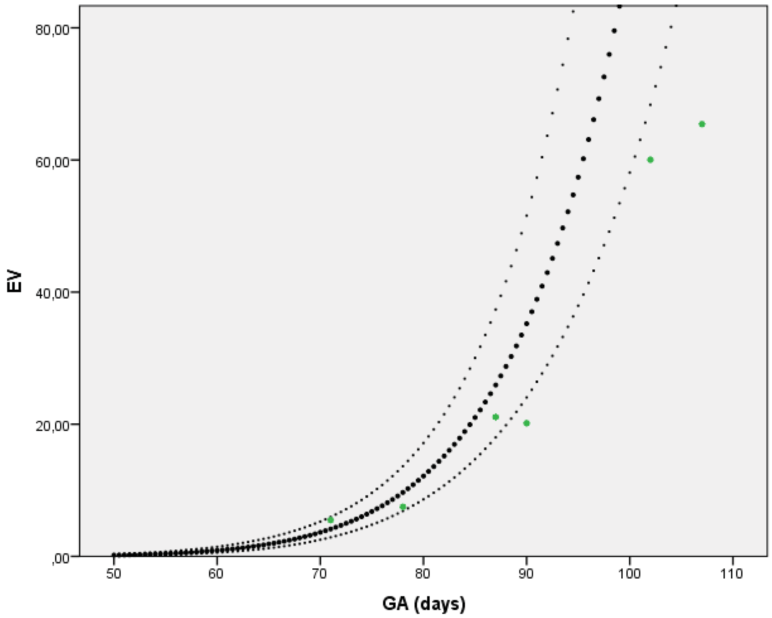
EV
GA (days)
80,00
60,00
40,00
20,00
,00
50
60
70
80
90
100
110

D

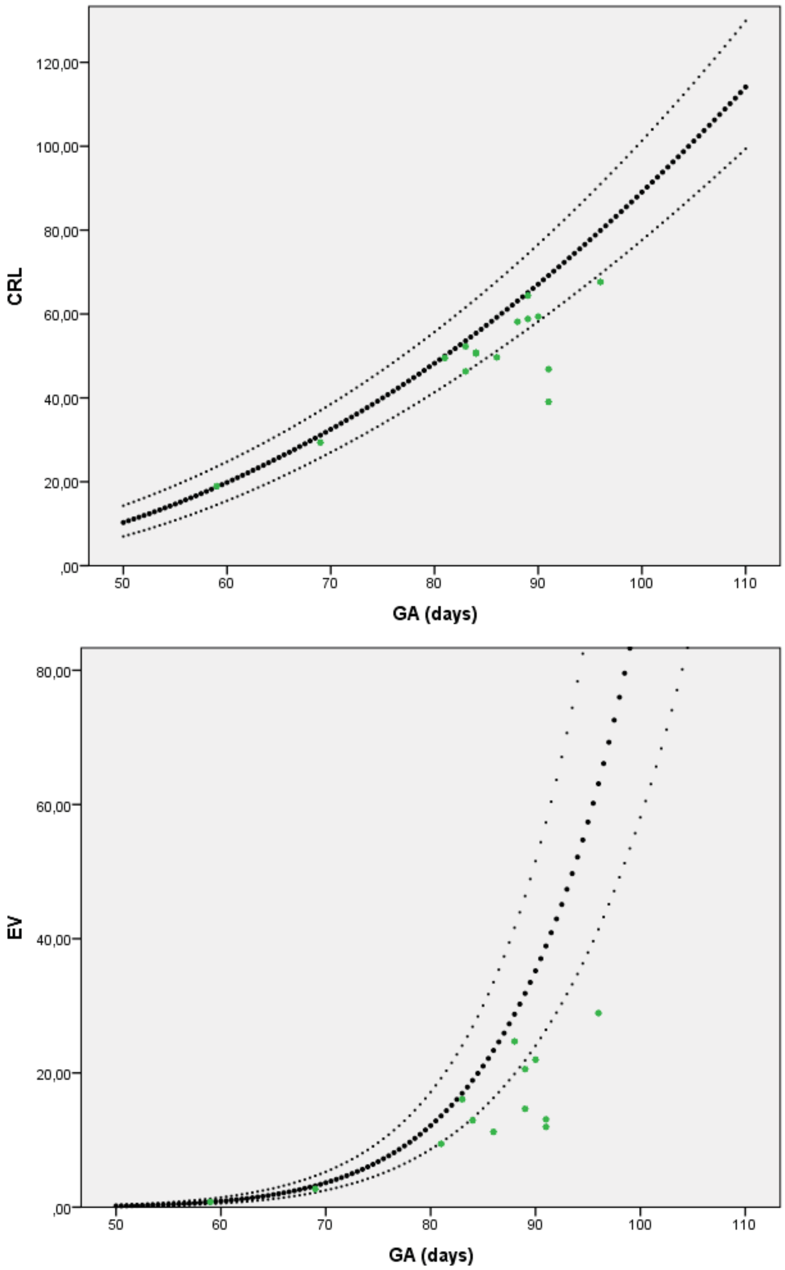
CRL
120,00
100,00
80,00
60,00
40,00
20,00
,00
50
60
70
80
90
100
110
GA (days)
EV
80,00
60,00
40,00
20,00
,00
50
60
70
80
90
100
110
GA (days)

E

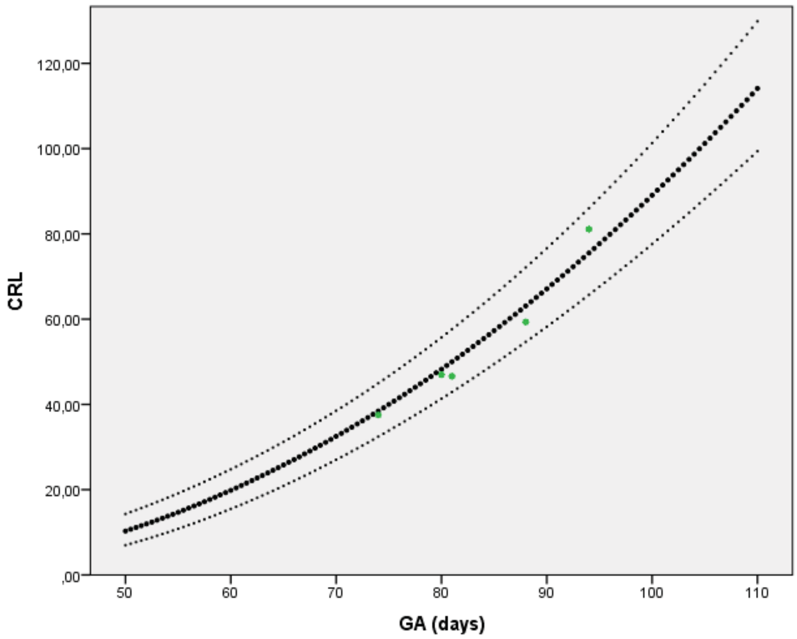
120,00
100,00
80,00
60,00
40,00
20,00
,00
CRL
50
60
70
80
90
100
110
GA (days)

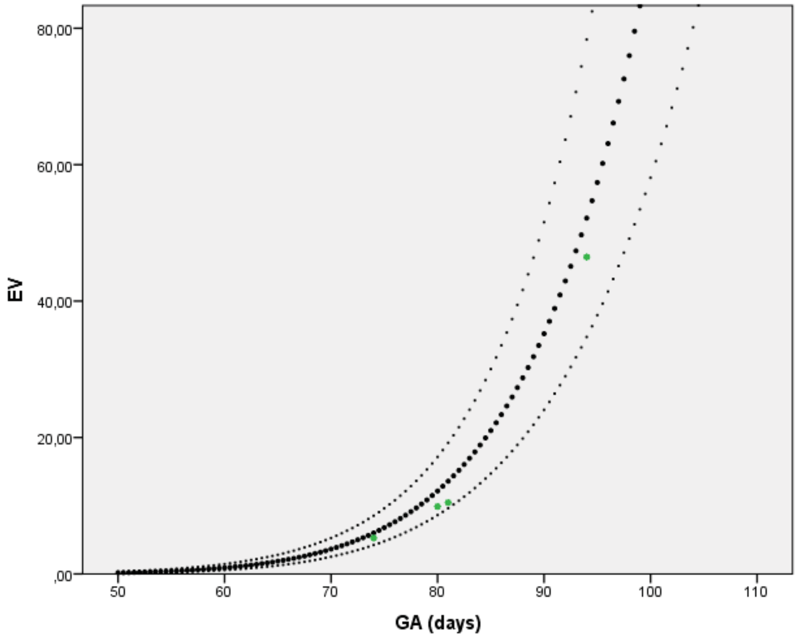
80,00
60,00
40,00
20,00
,00
EV
50
60
70
80
90
100
110
GA (days)

F

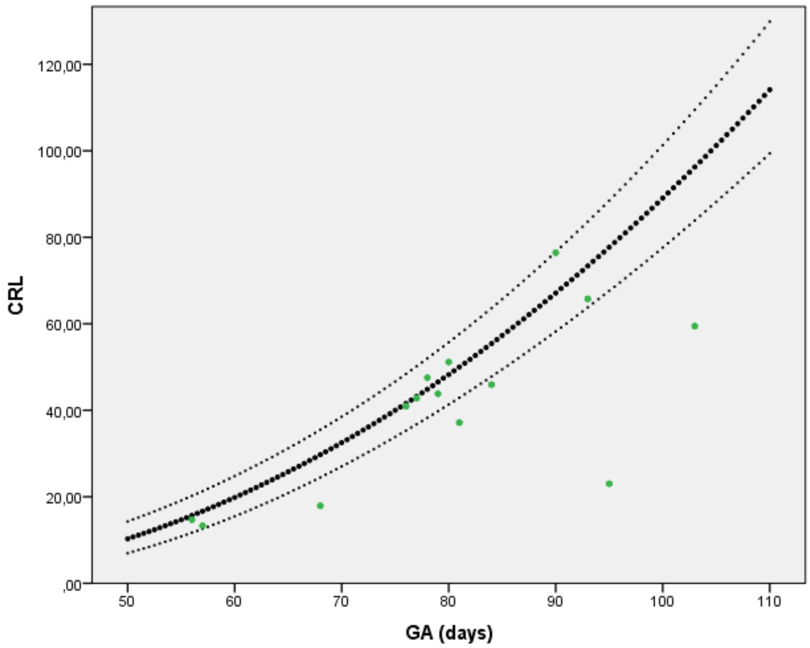
CRL
GA (days)
120,00
100,00
80,00
60,00
40,00
20,00
,00
50
60
70
80
90
100
110

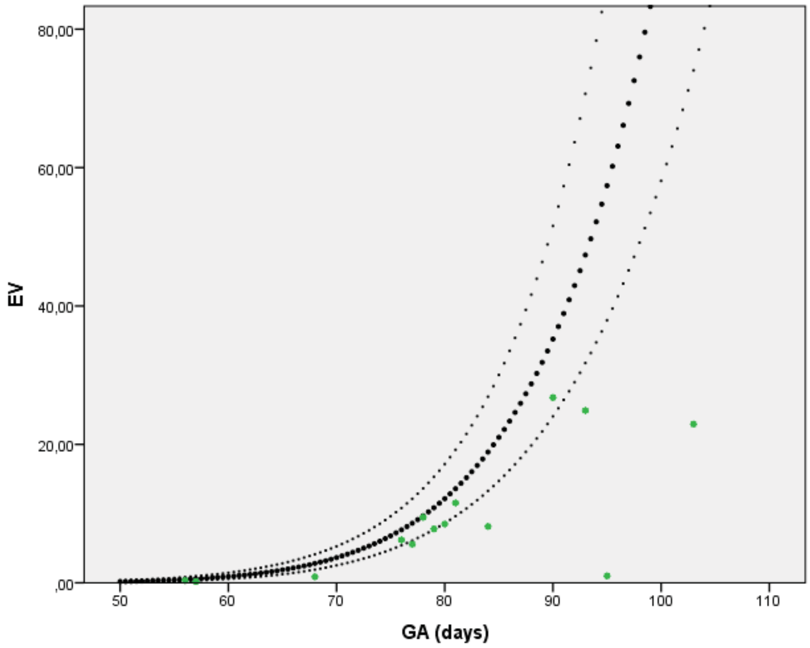
EV
GA (days)
80,00
60,00
40,00
20,00
,00
50
60
70
80
90
100
110

G

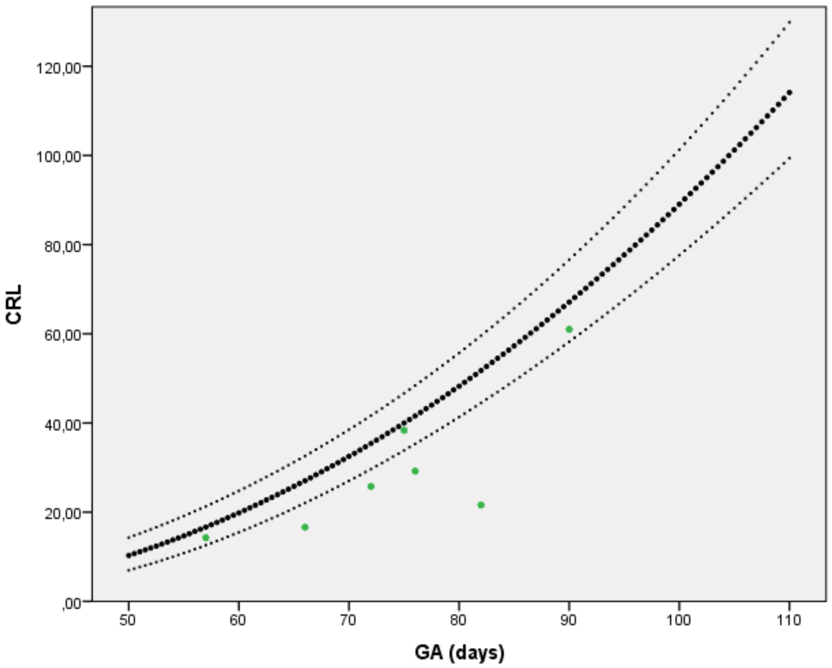
120,00
100,00
80,00
60,00
40,00
20,00
,00
CRL
50
60
70
80
90
100
110
GA (days)

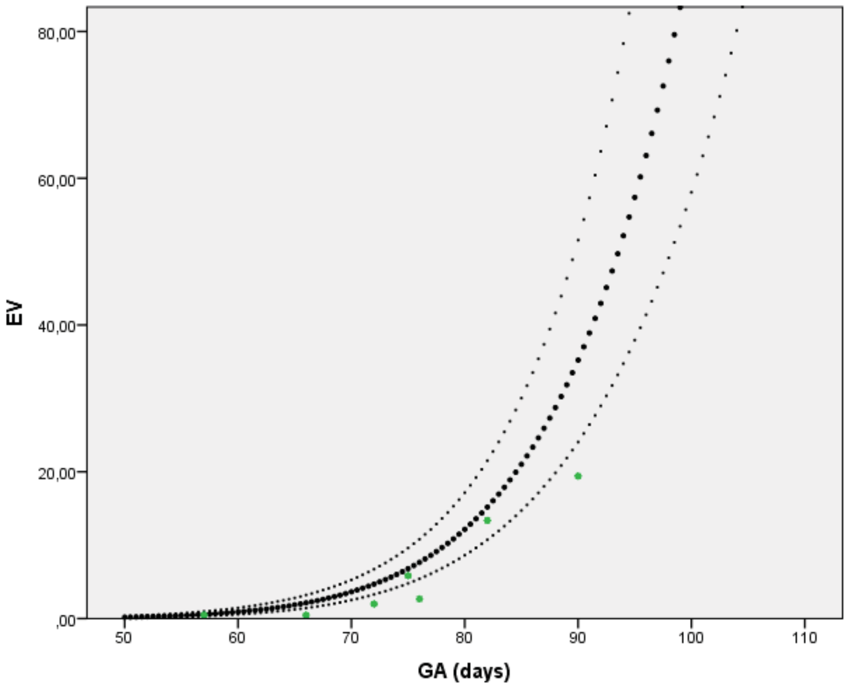
80,00
60,00
40,00
20,00
,00
EV
50
60
70
80
90
100
110
GA (days)
